# 可切除非小细胞肺癌新辅助免疫治疗研究进展

**DOI:** 10.3779/j.issn.1009-3419.2024.102.06

**Published:** 2024-02-20

**Authors:** Chang QI, Panwen TIAN, Weimin LI

**Affiliations:** ^1^610041 成都，四川大学华西医院呼吸与危重症医学科，呼吸和共病全国重点实验室，精准医学四川省重点实验室（齐畅，田攀文，李为民）; ^1^Department of Pulmonary and Critical Care Medicine, State Key Laboratory of Respiratory Health and Multimorbidity, Precision Medicine Key Laboratory of Sichuan Province, West China Hospital, Sichuan University, Chengdu 610041, China; ^2^610041 成都，四川大学华西医院呼吸健康研究所，疾病分子网络前沿科学中心，呼吸和共病研究院（李为民）; ^2^Institute of Respiratory Health, Frontiers Science Center for Disease-related Molecular Network, West China Hospital, Sichuan University, Chengdu 610041, China; ^3^610041 成都，四川大学华西医院中国医学科学院创新单元（李为民）; ^3^The Research Units of West China, Chinese Academy of Medical Sciences, West China Hospital, Chengdu 610041, China; ^4^610041 成都，四川大学华西医院肺癌中心（田攀文）; ^4^Lung Cancer Center/Lung Cancer Institute, West China Hospital, Sichuan University, Chengdu 610041, China

**Keywords:** 肺肿瘤, 新辅助治疗, 免疫检查点抑制剂, 围手术期, 可切除非小细胞肺癌, Lung neoplasms, Neoadjuvant therapy, Immune checkpoint inhibitors, Perioperative period, Resectable non-small cell lung cancer

## Abstract

近年来，免疫检查点抑制剂（immune checkpoint inhibitors, ICIs）对晚期非小细胞肺癌（non-small cell lung cancer, NSCLC）患者预后的改善已成为共识，越来越多的临床研究也逐渐证明了免疫治疗对于可切除NSCLC患者的重要价值。然而，目前关于新辅助治疗背景下免疫联合策略的探索、治疗相关副作用、预后生物标志物等问题仍存在争议。本文综述了可切除NSCLC患者新辅助免疫治疗的最新进展，引发了新的思考，并讨论了其在临床应用中的优势及挑战。

## Abstract (en)

In recent years, there has been a consensus regarding the enhancement of prognosis in patients with advanced non-small cell lung cancer (NSCLC) through the utilization of immune checkpoint inhibitors (ICIs). Numerous clinical studies have also demonstrated the substantial value of immunotherapy for resectable NSCLC patients. Nevertheless, there remain controversies surrounding the exploration of immune combination strategies, treatment-related side effects, prognostic biomarkers, as well as other issues in the neoadjuvant therapy setting. Consequently, this article presents a comprehensive overview of the recent advancements in neoadjuvant immunotherapy for resectable NSCLC, stimulating fresh perspectives and delving into its merits and challenges in clinical application.

随着肺癌筛查意识的普及和计算机断层扫描（computed tomography, CT）在高风险人群中的广泛应用，早期肺癌的检出率明显提升^[1]^。尽管手术是肺癌的首选治疗方式，然而早期非小细胞肺癌（non-small cell lung cancer, NSCLC）患者根治术后复发率高，且随着分期增加，只有25%-30%的NSCLC患者可以在术后彻底清除病灶，故围手术期全身治疗十分必要^[2]^。在过去的20年里，含铂术后辅助化疗被推荐作为II-IIIA期NSCLC患者的标准治疗方式^[3,4]^，然而研究^[3,5⇓-7]^发现，与单纯手术相比，尽管辅助化疗组在无病生存期（disease-free survival, DFS）的观察终点上有显著优势，但5年生存率的增加十分有限（约5%）。新辅助化疗模式的出现进一步延长了IB-IIIA期肺癌患者的总生存期（overall survival, OS）和5年DFS，但总体获益仍有待提高，可切除NSCLC患者的长期生存是研究者们关注的热点^[8]^。免疫检查点抑制剂（immune checkpoint inhibitors, ICIs）已在驱动基因突变阴性的晚期NSCLC患者的全身抗肿瘤治疗中广泛应用，显著提高了生存率^[9]^。而针对可切除NSCLC患者，许多新辅助免疫治疗的研究也已推进到临床III期，证明了围手术期免疫治疗的前景，见表1^[10⇓⇓⇓⇓-15]^。因此，本文综述了新辅助免疫治疗的热点研究，总结研究结果，为可切除NSCLC患者的围手术期临床诊疗提供参考。

**表1:** 可切除NSCLC新辅助免疫治疗III期临床研究进展（2023年）

Clinical trials	n	Conference	Stage	Exclude patients with oncogenic drivers	Regimen (Neoadjuvant/Adjuvant)	MPR	pCR	Surgical feasibility	Survival data
KEYNOTE-671^[10]^	797	ASCO	II-IIIB (N2)	No	Pembrolizumab+Chemo/Pembrolizumab	30.2%	18.1%	82.1%	mEFS (NR vs 17.0 mon, HR=0.58)
Neotorch^[11]^	404	ASCO	II-III	Yes	Toripalimab+Chemo/Toripalimab+Chemo (1 cycle) and Toripalimab (13 cycles)	48.5%	24.8%	82.2%	mEFS (NR vs 15.1 mon, HR=0.40)
AEGEAN^[12]^	802	AACR	IIA-IIIB (N2)	No	Durvalumab+Chemo/Durvalumab	33.3%	17.2%	80.6%	mEFS (NR vs 25.9 mon, HR=0.68)
RATIONALE-315^[13]^	453	ESMO	II-IIIA	Yes	Tislelizumab+Chemo/Tislelizumab	56.0%	41.0%	84.1%	-
CheckMate 77T^[14]^	461	ESMO	IIA-IIIB (N2)	Yes	Nivolumab+Chemo/Nivolumab	35.4%	25.3%	77.0%	mEFS (NR vs 18.0 mon, HR=0.58)
CheckMate 816^[15]^	358	ELCC	IB-IIIA	Yes	Nivolumab+Chemo	36.9%	24.0%	83.0%	mEFS (NR vs 21.1 mon, HR=0.68), EFS at 3-yr (57.0% vs 43.0%), OS at 3-yr (78.0% vs 64.0%)

NSCLC: non-small cell lung cancer; Chemo: chemotherapy; NR: non-responder; OS: overall survival; mEFS: median event-free survival; PFS: progression-free survival; MPR: major pathologic response; pCR: pathologic complete response; HR: hazard ratio; AACR: American Association for Cancer Research; ASCO: American Society of Clinical Oncology; ESMO: European Society for Medical Oncology; ELCC: European Lung Cancer Congress.

## 1 新辅助免疫治疗

新辅助免疫治疗在可切除NSCLC患者中作用的原理主要包括增强对微转移灶的靶向作用和术前降低肿瘤负荷。一方面，新辅助免疫治疗可以早期攻击微转移并减轻肿瘤免疫抑制性^[16⇓⇓-19]^。ICIs通过阻断免疫检查点结合带来的免疫逃逸，增加绝对淋巴细胞计数（absolute lymphocyte count, ALC），恢复耗竭CD8^+^ T细胞的功能，诱导效应T细胞的表型和功能改变，增强抗肿瘤免疫反应^[20]^。且初诊时肿瘤细胞未暴露于治疗相关的选择压力，拥有较高的肿瘤突变负荷（tumor mutational burden, TMB）和有限的异质性，此时免疫治疗将得到最大获益^[21,22]^。另一方面，新辅助治疗带来的肿瘤退缩和淋巴结降期，可以降低手术难度，提高R0切除率，甚至改变广泛切除的手术方案。

尽管以抗程序性死亡受体1（programmed cell death 1, PD-1）/程序性死亡配体1（programmed cell death ligand 1, PD-L1）药物为代表的ICIs在抗肿瘤治疗中取得了巨大成功，但仍存在部分患者对其耐药。针对细胞毒性T淋巴细胞相关蛋白4（cytotoxic T lymphocyte-associated antigen-4, CTLA-4）、T细胞免疫球蛋白黏蛋白3（T cell immunoglobulin domain and mucin domain-3, TIM-3）、淋巴细胞活化基因3（lymphocyte activation gene 3, LAG-3）、T细胞免疫球蛋白和ITIM结构域（T cell immunoreceptor with Ig and ITIM domain, TIGIT）、自然杀伤（natural killer, NK）细胞表面受体NKG2A、CD73等新型免疫检查点药物的临床研究也在逐步应用于NSCLC围手术期的治疗^[23]^。新型免疫药物与PD-1/PD-L1抗体的组合可以协同激活T细胞，进而恢复机体细胞免疫的杀伤作用，更高效地抑制肿瘤进展。

由于DFS和OS等指标的获得需要多年随访，临床研究^[24,25]^中往往采用病理完全缓解（pathologic complete response, pCR）或主要病理缓解（major pathologic response, MPR）作为替代疗效评估终点，二者已被证明与生存结果密切相关，其应用可以缩短治疗方案的评价时间。Liu等^[24]^通过动物模型发现，新辅助免疫治疗与手术切除的时间间隔过长或过短均会影响疗效，并且延长治疗周期并不一定提高生存获益。参照新辅助化疗，目前新辅助免疫治疗临床研究的术前用药时长在2-4个周期不等，单药新辅助治疗多采用2个周期，联合疗法多采用3-4个周期。

## 2 新辅助免疫单药治疗

CheckMate 159^[26]^是评估新辅助免疫单药治疗NSCLC安全性和可行性的首批试点研究之一，开启了围手术期免疫治疗新时代。这项研究评估了21例术前接受纳武利尤单抗治疗的I-IIIA期患者，45%的患者出现MPR，10%的患者达到pCR。而既往研究^[27⇓-29]^中，新辅助化疗的MPR大多在20%以下。而LCMC3研究^[30,31]^纳入了181例表皮生长因子受体（epidermal growth factor receptor, EGFR）/间变性淋巴瘤激酶（anaplastic lymphoma kinase, ALK）突变阴性的IB-IIIB期可切除NSCLC患者，术前接受2次阿替利珠单抗治疗，有获益的患者术后继续服用阿替利珠单抗≤12个月。结果显示该方案耐受性良好，无安全性问题，但MPR率仅为21%，pCR率为7%。此外，一些小样本研究也证实了信迪利单抗、帕博利珠单抗、阿替利珠单抗在NSCLC患者单药新辅助治疗中的抗肿瘤活性良好，且安全可耐受，MPR率分别为40.5%^[32]^、40%^[33]^和57%^[34]^。然而，不同免疫药物的研究中MPR率差异较大，其病理反应率和生存结果需要在更大规模的临床试验中进一步评估。

## 3 新辅助免疫联合治疗

### 3.1 新辅助免疫双药治疗

Reuss等^[35]^对9例可切除的IB（≥4 cm）-IIIA期NSCLC患者，在计划手术前6周予以纳武利尤单抗+伊匹木单抗（CTLA-4抗体），术前约4、2周时再次给予纳武利尤单抗。所有患者均接受了计划剂量的新辅助治疗，并完成手术。然而，9例患者中有6例（67%）出现治疗相关不良事件（treatment related adverse events, TRAEs），并且3例（33%）TRAEs≥3级。

随机对照II期临床研究NEOSTAR^[36]^同样探索了纳武利尤单抗联合伊匹木单抗双药免疫新辅助治疗在可切除NSCLC患者中的疗效。在每组44例入组患者中，双免联合组客观缓解率（objective response rate, ORR）为19%，MPR率为33%，pCR率为38%；而纳武利尤单药组ORR为22%，MPR率达到17%，pCR率达到10%。双药连用并未显著提高疗效，但存在免疫相关不良事件（immune-related adverse events, irAEs）增加的风险^[37]^。而在后续联合化疗的进一步研究^[38]^中，结果表明单药免疫联合化疗组MPR率为32.1%，双免疫联合化疗组MPR率为50%。在EGFR/ALK突变阴性的患者亚群中，两组的MPR率分别为41.2%（7/17）和62.5%（10/16）。利用单细胞测序和多重荧光免疫组化等多组学技术分析免疫细胞群的表型及浸润情况，发现效应记忆CD8^+^ T、B细胞以及三级淋巴结构在双免疫+化疗队列中显著增加。新辅助纳武利尤单抗+伊匹木单抗联合化疗的治疗方式增强了可切除NSCLC的病理反应，且双重ICIs增强抗肿瘤免疫活性并减轻免疫抑制表型，总体治疗安全且耐受。

此外，NEOPREDICT-Lung研究^[39]^探究了LAG-3抑制剂瑞拉利单抗联合纳武利尤单抗在IB-IIIB期NSCLC患者新辅助免疫治疗的作用。结果显示，43 d内手术切除率100%，57例患者获得R0切除。方案安全可控，没有增加并发症的风险。而NeoCOAST研究^[40]^是一项随机、多药、平台型临床试验，比较了度伐利尤单抗、度伐利尤单抗联合抗CD73单克隆抗体奥来鲁单抗、度伐利尤单抗联合NKG2A抑制剂莫那利珠单抗和度伐利尤单抗联合信号转导因子和转录激活因子3（signal transducer and activator of transcription 3, STAT3）抑制剂Danvantisen几种新辅助免疫治疗方式的疗效和安全性。84例I-IIIA期可切除NSCLC患者被随机分配至4个治疗组中，以MPR为主要终点，单药治疗MPR率为11.1%，度伐利尤单抗联合抗CD73组MPR率为19%，度伐利尤单抗联合抗NKG2A组MPR率为30.0%，而度伐利尤单抗联合抗STAT3组MPR率为31.3%，所有双免疫组MPR率都高于单药免疫组；就安全性而言，各组患者TEAEs发生率无显著差异。

对于新辅助免疫治疗，多种免疫通路联合治疗效果可能优于ICIs单药治疗，但不同药物的疗效及安全性存在差异，针对新型免疫药物正在开展的NeoCOAST-2、CheckMate 816研究中关于纳武利尤单抗联合伊匹木单抗双药免疫的探索性分析结果等或许将会为这些药物的临床应用提供更多数据支持^[41]^。

### 3.2 新辅助免疫联合化疗

#### 3.2.1 无驱动基因突变的NSCLC

Luis A.Godoy团队^[42]^总结了非癌基因驱动的可切除NSCLC新辅助免疫联合化疗的新数据，超过80%的患者接受新辅助免疫联合化疗后能够完全切除肿瘤，并且ICIs联合化疗相较于ICIs单药在病理反应上更好。而SAKK 16/14研究^[43]^探索了在顺铂和多西他赛的标准新辅助化疗基础上序贯使用度伐利尤单抗的疗效，在纳入的68例IIIA期患者中，MPR率达到60%。

CheckMate 816研究^[15,44]^评估了肿瘤直径≥4 cm或淋巴结阳性IB-IIIA期NSCLC患者（排除EGFR/ALK）纳武利尤单抗联合铂类双药化疗的预后。研究发现，与只接受化疗的患者相比，纳武利尤单抗联合化疗的患者手术切除淋巴结中的残余肿瘤细胞更少，其pCR率显著提高，无事件生存期（event-free survival, EFS）与3年OS显著延长。这是第一个获得美国食品药品监督管理局（Food and Drug Administration, FDA）批准的新辅助免疫治疗方式，尽管适用于任何PD-L1表达状态的可切除NSCLC患者，但IIIA期或PD-L1表达≥50%的患者获益最大，PD-L1表达<1%或IB-II期患者从该方案中获益较少，而PD-L1表达为1%-49%的亚组则没有明确的获益，高PD-L1表达组可能是该方法的最佳适用人群。在手术结果方面，83%的纳武利尤单抗联合化疗组患者和75%的单纯化疗组患者进行了手术。延迟手术的患者比例（21% vs 18%）与因不良事件延迟手术的比例（4% vs 7%）在两组间相似。中位随访29.5个月时，联合组中微创手术、开胸、肺叶切除术或全肺切除术各亚组均未达到中位EFS。总体TRAEs和严重（3-4级）TRAEs在两组中相似，irAEs发生率较低，在纳武利尤单抗联合化疗组中仅出现2例（1.1%）。根据缩瘤效果和安全性综合考量，目前免疫治疗联合化疗可能是潜在的最佳新辅助模式。

#### 3.2.2 存在驱动基因突变的NSCLC

吴一龙教授团队^[45]^首先探究了存在癌基因突变的可切除NSCLC新辅助免疫联合化疗的临床可行性。研究入组的40例患者包含19例EGFR突变患者、9例KRAS突变患者和12例其他突变患者。在所有患者中，新辅助免疫联合化疗的MPR率为37.5%，pCR率为12.5%。将EGFR突变亚组与CTONG1103研究中EGFR突变队列数据整合，对化疗（GC方案）、靶向治疗（厄洛替尼）和免疫联合化疗3种新辅助治疗方式进行后续分析，发现免疫联合化疗组的疗效最为优越。Shu等^[34]^关于新辅助阿替利珠单抗联合化疗的II期临床研究，纳入了4例EGFR突变患者，其中2例患者（L858R和L858R/S768I突变）达到pCR，其余2例患者（外显子20插入和外显子19缺失）则反应不佳。此外，KEYNOTE-671、AEGEAN等临床研究^[10,12]^也提供了一些驱动基因阳性患者的治疗数据。AEGEAN^[12]^在其研究初期纳入了EGFR突变患者，其中26例接受度伐利尤单抗联合化疗的新辅助治疗，然而，这部分人群的pCR率为3.8%，MPR率为7.7%，且EFS也大幅降低，治疗获益远远低于研究中驱动基因突变阴性患者。

总的来说，对于存在驱动基因突变的NSCLC，靶向治疗是更有针对性的选择，且明确驱动基因十分必要。NAUTIKA1是一项正在开展的多种靶点靶向新辅助治疗的研究^[46]^，涵盖ALK、ROS1、NTRK、BRAF V600E、RET和KRAS多种突变的患者，初步数据显示，在9例ALK突变患者中，靶向治疗后6例可达MPR（66.7%），3例可达pCR，这提示我们ALK突变患者可能更适合术前的新辅助治疗。但对存在TP53、STK11等常见共突变、尚无靶向药可用或无法完成基因检测的患者，化疗联合免疫的新辅助治疗或许是可以尝试的新方向。

### 3.3 新辅助免疫联合抗血管生成药物

化疗是ICIs最常见的联用方案，但对于含铂化疗反应不佳或副作用较大的患者，新辅助免疫联合治疗方案还没有被充分探索。ICIs联合抗血管生成药物的方案在晚期NSCLC患者中的有效性和安全性已被报道，这为早期患者新辅助治疗的发展提供参考^[47,48]^。

最近的一些临床研究也评估了新辅助免疫联合抗血管生成治疗的疗效。EAST ENERGY是一项II期单臂开放标签的临床研究^[49]^，旨在评估2个周期新辅助帕博利珠单抗联合雷莫卢单抗治疗可切除的PD-L1阳性NSCLC患者的有效性和安全性。研究中MPR率为50%，pCR率为25%。中位随访23.6个月时，中位无复发生存期（recurrence free survival, RFS）和中位OS均尚未达到。除此之外，安罗替尼联合帕博利珠单抗的新辅助治疗临床研究也正在开展（NCT04762030）。抗血管药物与ICIs的协同抗肿瘤作用或许可以弥补单药免疫在术前缩瘤效果上的不足。

## 4 “新辅助+辅助”免疫全程治疗

新辅助免疫治疗重塑了驱动基因突变阴性的早期肺癌的治疗格局。然而，目前尚不清楚新辅助治疗是否优于辅助治疗，关于围手术期管理可能会引起进一步的混淆。在过去几年中，关于围手术期全程治疗方法的临床研究越来越多，并且占正在进行的大型随机对照临床研究的大多数。围手术期治疗策略结合术前和术后给药，将ICIs与化疗相结合，在手术前最大限度地缩小肿瘤和控制转移趋势；基于维持手术结果和消除潜在微小残留病变（minimal residual disease, MRD）的理由，单药ICIs也在辅助治疗中序贯使用。

NADIM是一项开放标签、多中心、单臂、II期研究^[50]^，纳入了可手术的46例IIIA期NSCLC患者，术前予以纳武利尤单抗+紫杉醇+卡铂3个周期新辅助治疗，术后接受为期1年的辅助纳武利尤单抗维持治疗。在接受手术的患者中，24个月的PFS率为77.1%，MPR率为83%，pCR率为71%，且3年OS率提升到了81.9%。纳武利尤单抗的“夹心饼”式围手术期治疗树立了NSCLC围手术期免疫治疗的一块重要里程碑。而2023年新英格兰报道的NADIM II研究^[51]^进一步弥补了NADIM未设置对照的缺点，并增加了IIIB期患者的入组。纳武利尤单抗组术前pCR率是对照组的5倍以上（37% vs 7%），手术率和R0切除率提高，24个月OS率较对照组提升了21.4%，死亡风险下降57%，实现了全方位的治疗获益。AEGEAN是首个报阳的围手术期免疫治疗III期研究^[12]^，对802例患者进行分组，术前予以度伐利尤单抗联合含铂化疗4个周期，术后予以度伐利尤单抗辅助治疗，已公布的数据显示，相较于仅接受术前新辅助化疗，度伐利尤单抗联合化疗可显著改善患者pCR和EFS获益。针对中国人群的Neotorch^[11]^旨在评估围手术期特瑞普利单抗或安慰剂联合化疗对EGFR/ALK野生型III期可手术II/III期NSCLC的疗效和安全性。目前公布的预后数据显示，无论患者PD-L1表达水平、组织学类型，特瑞普利单抗联合化疗组的MPR率和pCR率均大幅提升（48.5% vs 8.4%; 24.8% vs 1.0%），中位EFS显著延长，疾病复发、进展或死亡风险下降60%，OS也显示出明显的获益趋势，且总体不良反应可控。该研究高度符合中国临床实践，为我国早期NSCLC患者的诊疗提供了真实世界数据。

2023年美国临床肿瘤大会（American Society of Clinical Oncology, ASCO）同样报告了KEYNOTE-671^[10]^的部分分析数据。继KEYNOTE-189和KEYNOTE-407之后，KEYNOTE-671把免疫联合含铂化疗的治疗方式从NSCLC晚期推向早期围手术期治疗，旨在评估术前帕博利珠单抗联合铂类化疗新辅助治疗+术后帕博利珠单抗辅助治疗的疗效与安全性。结果发现，帕博利珠单抗组pCR率、MPR率均显著高于安慰剂组，且拥有更长的EFS及OS。而欧洲肿瘤内科学会（European Society for Medical Oncology, ESMO）则公布了RATIONALE-315^[13]^的研究结果。该研究纳入了453例中国患者，评估可切除II-IIIA期NSCLC患者接受替雷利珠单抗联合含铂双药化疗“新辅助+辅助”免疫全程治疗的疗效和安全性，MPR和EFS双终点均得到阳性结果，且pCR率高达41%。同时，CheckMate 77T研究^[14]^在CheckMate 816研究纳武利尤单抗联合化疗（NIVO+化疗）新辅助方案的基础上，术后增加纳武利尤单抗辅助免疫治疗，结果表明，NIVO+化疗/NIVO组中位EFS显著延长（NR vs 18.4个月），pCR率与MPR率获益同样十分明显（25.3% vs 4.7%; 35.4% vs 12.1%），进一步降低了患者的复发风险。且无论是否达到pCR或MPR，NIVO+化疗/NIVO组比化疗/安慰剂组均更有EFS获益趋势。未来各项大型临床研究的后续数据更新，或将进一步提升NSCLC患者围手术期全程免疫治疗的地位。

## 5 新辅助免疫治疗的问题与挑战

### 5.1 免疫耐受性与毒副作用

新辅助免疫治疗的一个潜在隐患是TRAEs，存在延迟手术和增加进展的风险。且因为早期癌症患者可能比晚期癌症患者有更强的免疫反应^[52]^，免疫治疗的副作用可能使完全切除病灶变得更具挑战性。截至目前，大多数研究的不良反应可控，严重药物毒副作用罕见。NEOSTAR^[53]^和Sintilimab^[32]^研究中各有1例患者术后出现治疗相关的致命的呼吸毒性。Takada等^[54]^总结了2017年1月1日至2023年7月27日发布的基于新辅助ICIs的全身治疗研究数据。发现导致手术取消最常见的原因是疾病进展，不良事件可能带来手术时机的延迟，但很少导致手术取消。在围手术期各并发症中，肺炎占比为0%-5.7%，脓胸为0%-5%，支气管胸膜瘘为0%-25%，心律失常为0%-30%，呼吸衰竭为0%-3.8%，血栓栓塞事件为0%-7.7%。尽管部分医生认为新辅助免疫治疗后手术难度存在一定程度的提升，但各研究均未得出围手术期并发症较对照组显著增加的结论^[26,30,36]^。根据现有的证据，可以初步确认，术前使用ICIs安全且耐受性良好。

“夹心饼”式围手术期免疫治疗模式的获益毋庸置疑，但具体适应人群还需进一步细化。从目前许多数据来看，新辅助治疗后达到pCR的患者接受术后辅助免疫治疗是获益的。但在以药物联合为主的新辅助方案中，是否存在过度治疗仍是需要考虑的问题。然而，真实世界的pCR判断标准不完善，MRD检测存在误差，可能需要对所有患者均采取围手术期全程免疫治疗模式以保障获益。至于未达到病理缓解的患者如何选择术后辅助方案，现有循证医学证据还很难给出准确答案。而另一方面，尽管新辅助免疫治疗组的手术率及R0切除率往往高于对照组^[14,44]^，但仍存在少部分患者疾病进展甚至失去治疗性手术机会。在LCMC3试验^[31]^中，181例患者中有13例（7.2%）在使用阿替利珠单抗期间由于临床或放射学疾病进展而未接受切除术。NEOSTAR研究^[53]^中，44例患者中有2例（4.5%）因疾病进展手术失败。如何延长新辅助免疫治疗失败的这部分患者的生存期，是围手术期治疗新的课题。

### 5.2 新辅助免疫治疗预后生物标志物

尽管关于免疫治疗标志物的研究始终在进行，但目前筛选新辅助免疫治疗获益人群仍很困难，且缺乏高质量的支持数据。与晚期NSCLC免疫治疗疗效评估相似，肿瘤细胞上的PD-L1高表达和肿瘤微环境（tumor microenvironment, TME）中表达PD-1的肿瘤浸润淋巴细胞（tumor infiltrating lymphocytes, TILs）是免疫治疗预后的有利因素。一项纳入了10项研究涉及461例NSCLC患者的meta分析^[55]^发现，与PD-L1表达<1%相比，PD-L1表达≥1%与患者更高的MPR率和pCR率相关。值得注意的是，50%作为PD-L1表达的临界值对MPR的预测效果优于1%。然而CheckMate 816^[15]^、KEYNOTE-671^[10]^、Neotorch^[11]^、CheckMate 77T^[14]^等多项临床研究发现，即使获益最明显的仍然是高表达人群，但无论PD-L1表达水平如何，患者均能从新辅助免疫治疗中获得pCR改善。这一结果也侧面反映了PD-L1并不能很好地区分出免疫治疗优势的人群。

循环肿瘤DNA（circulating tumor DNA, ctDNA）已在多个癌种表现出潜在预测价值，有望指导肺癌的临床诊疗；实现ctDNA清除也被认为是深化缓解、减少复发的重要先决条件^[56]^。CheckMate 816研究^[44]^以ctDNA清除率作为探索性研究终点，接受纳武利尤单抗联合化疗的24例患者的ctDNA清除率高于接受单纯化疗的19例患者（56% vs 35%），且与没有ctDNA清除的患者相比，存在ctDNA清除的患者EFS更长，pCR率更高。2023年ESMO会议上，AEGEAN研究^[57]^也更新了ctDNA相关数据，解析ctDNA清除率与pCR或MPR的关系。在每个治疗节点采集患者血样进行分析，发现与未达到pCR/MPR的患者相比，实现pCR/MPR的患者ctDNA变异等位基因频率（variant allele frequency, VAF）显著更低。基于度伐利尤单抗的围手术期方案能够通过提高ctDNA清除率，进而深化可切除NSCLC的缓解水平，提示ctDNA清除率可作为潜在的早期反应生物标志物。然而，与转移性NSCLC患者相比，早期NSCLC患者的血浆ctDNA水平较低，且目前大多数ctDNA分析方法对患者治疗期间和治疗后MRD检测缺乏足够的灵敏度，并且具有滞后性且成本较高^[58]^。研发高灵敏度的血液检测方式来定义全身MRD对肿瘤患者预后的预测十分必要^[59,60]^。

肿瘤组织与外周血中的免疫微环境也被认为是免疫治疗疗效的生物标志物。早期研究^[61⇓-63]^表明，CD4^+^和CD8^+^ T细胞在肿瘤组织中的浸润与较好的免疫治疗反应相关，而血液中淋巴细胞水平低则可能增加死亡风险。CheckMate 159研究^[26]^发现接受纳武利尤单抗新辅助治疗后达到MPR的患者治疗后CD8^+^ T细胞浸润水平高于治疗前，提示T细胞富集可能是一个潜在的生物标志物。Ig样转录因子2（Ig-like transcript 2, ILT2）、白细胞介素（interleukin, IL）-6、IL-8、高水平干扰素（interferon, IFN）-γ等活性因子也被证明可能与新辅助免疫治疗MPR、OS相关^[26,31]^。此外，虽然TMB与ICIs的疗效和预后之间的关系在接受免疫治疗的晚期NSCLC患者中更为密切，但LCMC3研究中高TMB的患者也观察到更好的病理反应，且与更好的PFS相关^[64,65]^。同源重组缺陷（homologous recombination defect, HRD）也可以预测NSCLC患者新辅助免疫治疗结果。肿瘤抑制基因在DNA损伤修复和同源依赖性重组途径中的突变在MPR患者中更为常见，这表明反应较好的患者可能发生HRD事件。一项对14例通过免疫治疗联合化疗达到MPR的NSCLC患者（3例腺癌和11例鳞状细胞癌）的全外泌体分析^[66]^显示，HRD的检测与对新辅助免疫治疗的反应增强有关，并且接受免疫治疗的HRD患者除了有大量通路改变外，TMB也更高，生存期更长^[66]^。

## 6 结论与展望

总的来说，新辅助免疫治疗的优势在于可以早期靶向肿瘤微转移，有望降低肿瘤分期，增加手术成功率并实现完全切除，是降低复发率、改善无驱动基因突变的可切除NSCLC患者预后的首选治疗方式。联合治疗相比于单药免疫治疗，可以达到更好的抗肿瘤效果，但需考虑过度诊疗带来的药物副作用。而新辅助+辅助的“夹心饼”免疫疗法是围手术期管理发展的大趋势与最优解，带来更加显著的生存获益。肿瘤组织的PD-L1表达水平作为新辅助免疫治疗的预后预测标志物效果欠佳，精准医学的发展和个体化诊疗需要更多高效能生物标志物的检测和探索^[42]^。期待更多临床研究探讨围手术期全身治疗方案的选择与优劣，优化生物标志物，以筛选优势人群，为提高患者的生存率提供临床参考。

**Competing interests**

The authors declare that they have no competing interests.
